# Turn Your Vision into Reality—AI-Powered Pre-operative Outcome Simulation in Rhinoplasty Surgery

**DOI:** 10.1007/s00266-024-04043-9

**Published:** 2024-05-22

**Authors:** Samuel Knoedler, Michael Alfertshofer, Siddharth Simon, Adriana C. Panayi, Rakan Saadoun, Alen Palackic, Florian Falkner, Gabriel Hundeshagen, Martin Kauke-Navarro, Felix H. Vollbach, Amir K. Bigdeli, Leonard Knoedler

**Affiliations:** 1 Division of Plastic Surgery, Brigham and Women’s Hospital, Harvard Medical School, Boston, MA USA; 2 Department of Plastic and Hand Surgery, Klinikum Rechts der Isar, Technical University of Munich, Munich, Germany; 3 Department of Oromaxillofacial Surgery, Ludwig-Maximilians University Munich, Munich, Germany; 4 Department of Hand-, Plastic and Reconstructive Surgery, Microsurgery, Burn Center, BG Center Ludwigshafen, University of Heidelberg, Ludwigshafen, Germany; 5 Department of Hand and Plastic Surgery, University of Heidelberg, Heidelberg, Germany; 6 Department of Plastic Surgery, University of Pittsburgh, Pittsburgh, PA USA; 7 Department of Surgery, Division of Plastic Surgery, Yale School of Medicine, New Haven, CT USA; 8 Department of Plastic, Hand and Reconstructive Surgery, University Hospital Regensburg, Regensburg, Germany

**Keywords:** Rhinoplasty, Nose reshaping, Artificial intelligence, Pre-operative simulation, Computer simulation, Generative adversarial networks

## Abstract

**Background:**

The increasing demand and changing trends in rhinoplasty surgery emphasize the need for effective doctor–patient communication, for which Artificial Intelligence (AI) could be a valuable tool in managing patient expectations during pre-operative consultations.

**Objective:**

To develop an AI-based model to simulate realistic postoperative rhinoplasty outcomes.

**Methods:**

We trained a Generative Adversarial Network (GAN) using 3,030 rhinoplasty patients’ pre- and postoperative images. One-hundred-one study participants were presented with 30 pre-rhinoplasty patient photographs followed by an image set consisting of the real postoperative versus the GAN-generated image and asked to identify the GAN-generated image.

**Results:**

The study sample (48 males, 53 females, mean age of 31.6 ± 9.0 years) correctly identified the GAN-generated images with an accuracy of 52.5 ± 14.3%. Male study participants were more likely to identify the AI-generated images compared with female study participants (55.4% versus 49.6%; *p* = 0.042).

**Conclusion:**

We presented a GAN-based simulator for rhinoplasty outcomes which used pre-operative patient images to predict accurate representations that were not perceived as different from real postoperative outcomes.

**Level of Evidence III:**

This journal requires that authors assign a level of evidence to each article. For a full description of these Evidence-Based Medicine ratings, please refer to the Table of Contents or the online Instructions to Authors www.springer.com/00266.

## Introduction

The global rhinoplasty market is booming, with an estimated value of USD 6.2 billion in 2020 and a projected annual growth rate of 6.5% for the next seven years [1]. In the US alone, plastic surgeons performed more than 350,000 rhinoplasties in 2022 [2].

Owing to the procedure’s widespread popularity, the complexity of rhinoplasty can often be underestimated. With various techniques available—each of which is customized for specific indications and patient cohorts—rhinoplasty is considered one of the most challenging procedures in the field of plastic surgery [3].

Artificial Intelligence (AI) has emerged as a versatile workhorse to facilitate a wide array of clinical algorithms [4–7]. Specifically, Generative Adversarial Networks (GANs) have been established as helpful tools for outcome simulation, although they are commonly based on pre-/postoperative patient images but not the individual patient’s desire and expectations [8]. However, despite the well-documented applicability of GAN in visualizing potential outcomes after plastic and esthetic surgery, no study has investigated the applicability in a rhinoplasty cohort using multi-surgeon patient populations and quantifiable outcomes [8, 9].

To fill this research gap, we aimed to utilize the computational capacity of AI to develop a GAN-powered outcome simulation for rhinoplasty candidates. To assess the authenticity of these AI-generated outcome simulations, we presented them along with real postoperative images to study participants and tasked them to indicate which image was AI-generated. Ultimately, this line of research may unlock untapped potential in managing pre-operative patient expectations and depicting realistic postoperative outcomes.

## Materials and Methods

### Basic Considerations of the Generative Adversarial Network

The Generative Adversarial Network (GAN) learns to create realistic postoperative images from pre-operative ones by training on numerous image pairs. It uses a discriminator network to refine its ability to generate convincing images, improving over time. This process aims to produce predictions indistinguishable from actual postoperative photographs through iterative training, enhancing the model’s plausibility in simulating surgical outcomes.

### Database Creation

Pre-operative and postoperative images of 3,030 rhinoplasty patients (1,015 females) were retrieved from an online image database (https://www.realself.com). This study involved information that was already publicly available and, therefore, did not require IRB approval. As GAN training requires a fixed image size, all images were cropped to a square shape and resized to 256 × 256 pixels, centered horizontally on the midpoint of the nasal dorsum. The GAN was trained on 2,575 image pairs (85%), while the remaining pairs (*n* = 455; 15%) were used for model validation.

### GAN Training

The GAN architecture employed in this study is an adaptation of “pix2pix” by Isola et al. [10]. A copy of pix2pix was obtained from GitHub (https://github.com/junyanz/pytorch-CycleGAN-and-pix2pix.git) and implemented in Google Colaboratory (https://www.colab.research.google.com), a cloud service for the remote execution of hardware-intensive code. The network was trained on an Nvidia Tesla P100 16GB GPU for 250,000 iterations, i.e., the full training set was processed by the GAN 181.4 times. All hardware was hosted by Google Colaboratory.

### Study Participants

Study participants were recruited from the online study platform Prolific (https://www.prolific.com). No specific inclusion or exclusion criteria were applied during participant selection to achieve a diverse pool that could adequately represent the broad population.

### Survey Conduction

Study participants were presented with a total of 30 image sets consisting of three images each: (i) real pre-operative patient image, (ii) real postoperative patient image, and (iii) AI-generated potential postoperative surgical outcome for the respective patient. The original pre-operative patient image was consistently displayed on the left of the image set, while the remaining two images were randomized and labeled with “Option A)” and “Option B).” Study participants were then asked to identify which option has been generated using AI. There was no time limit for determining AI versus real patient images.

**Fig. 1:**
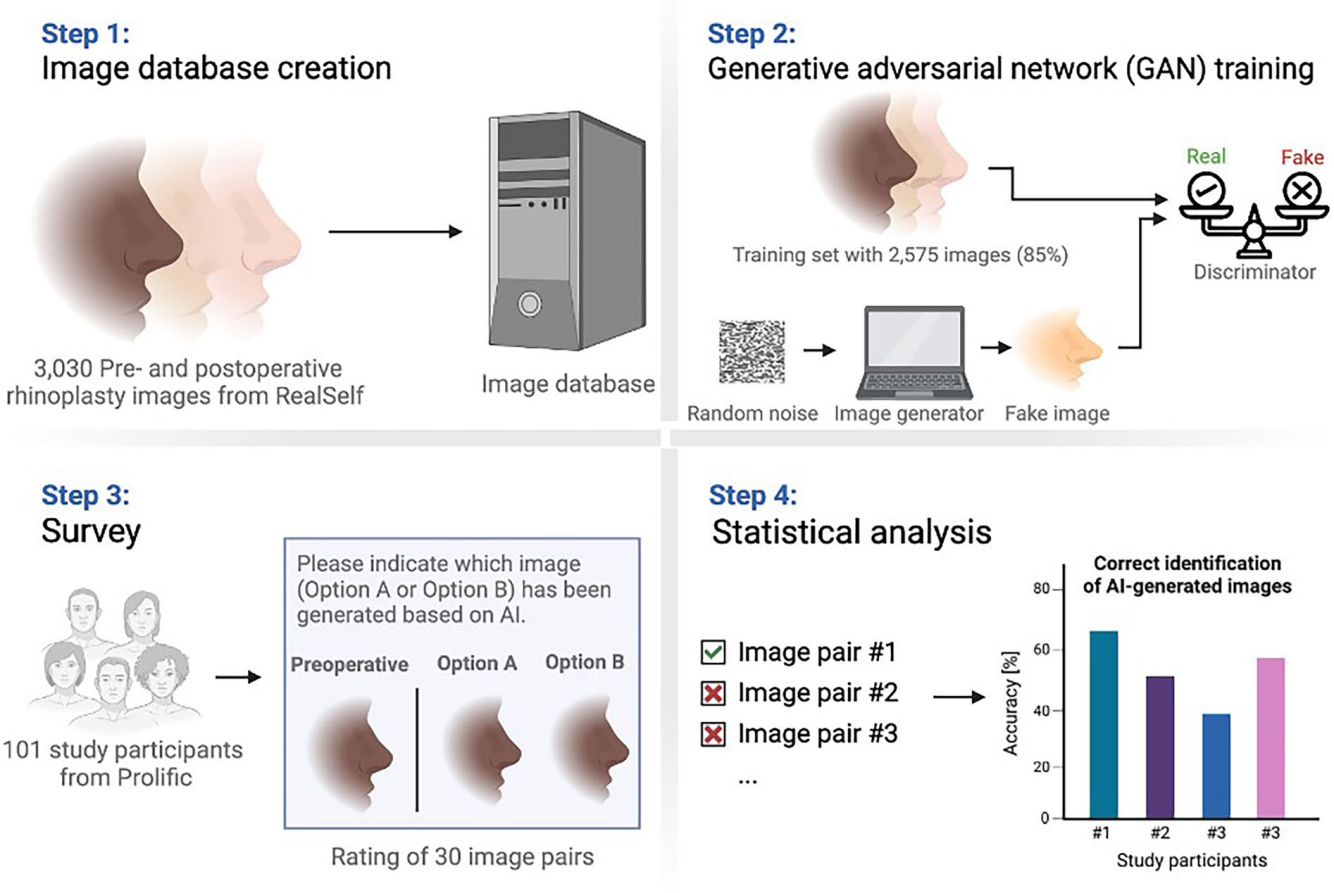
We subdivided the development and validation process into four key steps, ranging from the image database through the GAN training and the Prolific survey to the statistical analysis of the survey outcomes

The structure of each survey item was as follows (Fig. 1):

“Please indicate which image (Option A or B) has been generated based on artificial intelligence. The preoperative image is on the left.:

[Set consisting of three images]

⋄ Option A

⋄ Option B”

### Statistical Analysis

Differences for the correct identification of AI-generated images between gender, experience in plastic and esthetic surgery, consideration of undergoing/having undergone plastic surgery, and age were calculated using the independent Student's *t* test. All statistical analyses were run using SPSS Statistics 25 (IBM, Armonk, NY, USA), and differences were considered statistically significant at a probability value of *p* < 0.05.

## Results

### Study Participants

A total of 101 study participants with a mean age of 31.6 ± 9.0 years were recruited from the online study platform Prolific. The study sample consisted of 48 males and 53 females. Ten percent (*n* = 10) of study participants indicated that they have had prior experience with plastic and esthetic surgery in their life (e.g., underwent surgery and/or worked in this field), while 90% (*n* = 91) reported no experience in this regard. A total of 34.7% (*n* = 35) have considered undergoing and/or have underwent plastic and esthetic surgery, whereas 65.3% (*n* = 66) have indicated that they have not.

### Survey Conduction

**Fig. 2:**
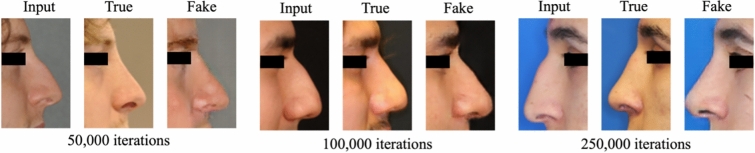
Over 50,000, 100,000, and 250,000 iterations, the GAN showed distinct improvements in the simulation of realistic postoperative outcomes

**Fig. 3:**
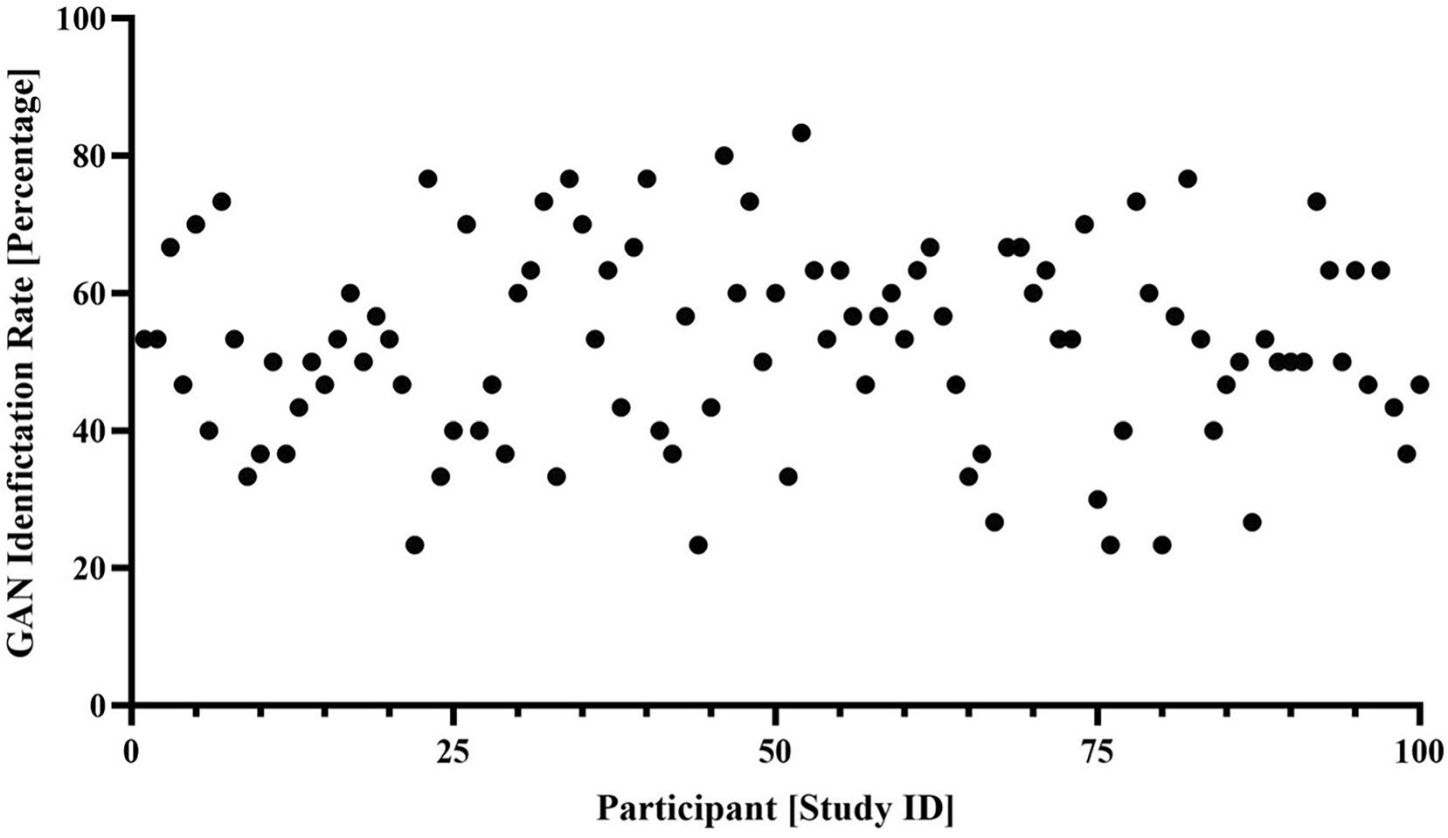
The overall GAN identification rate was 52.5 ± 14.3%, with moderate interindividual differences in GAN identification

The GAN-generated image was correctly identified in approximately half of all cases (52.5 ± 14.3%; 1,591/3,030; Figs. 2 and 3). On average, male study participants correctly identified the GAN-generated image in 55.4 ± 14.4% versus female study participants in 49.6 ± 13.7%, with *p* = 0.04.

There was no statistically significant difference between study participants with or without experience in plastic and esthetic surgery (*p* = 0.26) or between study participants who had considered undergoing or had undergone plastic and esthetic surgery versus those who had not (*p* = 0.72). Furthermore, when comparing younger versus older study participants (i.e., below and above the mean age), no statistically significant difference was found (*p* = 0.82). The average processing time per image set (i.e., the time between uploading the pre-operative image and generating the postoperative simulation) was 56 ± 11.8 ms. The development costs amounted to USD 321.60 for the Prolific human examination service.

## Discussion

In this study, we aimed to develop a GAN-driven outcome simulator to visualize postoperative results based on pre-operative images, thus paving the way toward more individualized patient education and counseling. We found that human evaluators correctly identified the GAN-generated image in 52.5% of all cases. The network’s average processing time per image set was 56 ms, while the total development costs amounted to USD 321.60.

GANs have shown promising potential in different medical fields [5]. However, prior studies on GANs for postoperative simulation have mainly relied on qualitative outcome descriptions, thus lacking quantifiable data points and human evaluation [8]. Further, the current research work on GAN-based rhinoplasty simulation focused on single-center and/or single-surgeon patient cohorts. For example, Bashiri-Bawil et al. [9] implemented profile photographs of 400 patients from a single-center database. While the authors reported an accuracy of 80%, defined as similarity measurement based on the Euclidian distance, the single-center study design may potentially introduce geographic bias. Overall, we aimed to overcome these limitations using a multi-surgeon database and quantifiable outcome measurements. In contrast to previous research, we also calculated the total development costs and the GAN processing to facilitate the development of future GAN models.

Using the current gold standard in AI-generated image examination (i.e., human examiner panel), we found that the 101 study participants correctly identified the GAN-generated image in only 52.5% [4]. In other words: In nearly half of all cases, the human raters were unable to distinguish simulations from actual postoperative images. This statistical coin toss generally underscores the computational power of our GAN. Therefore, the herein presented GAN-powered simulator substantiates not only GAN’s principal practicality and utility in outcome modeling but also marks a step forward in tomorrow’s implementation of AI-driven technologies in pre-operative patient counseling.

Our GAN was trained with input images derived from an online image database. So far, there is no scientific consensus to standardize image databases for GAN (and other AI-based software) training. Accordingly, different approaches are currently under investigation to optimize the data input and improve GAN performance. We accessed an online image database to extract pre-operative and postoperative images from 3,030 rhinoplasty patients. The online image database provides an open-access resource and image database with about 10 million monthly users [11], offering unbiased costs and procedure information with authentic patient images. In this context, it is worth mentioning that out of 55,968 (as of September 2023) rhinoplasty photographs available on the online image database, Inc. 44,657 (as of September 2023) showed the nasal side profile, which is one of the key perspectives included in standardized rhinoplasty photography [12]. Still, further studies are needed to define the optimal data source for training GAN and AI outcome simulators. In addition, a universally applicable image format and processing pattern should be established to effectively streamline future research.

The study’s use of GANs produces average surgical outcomes for patient consultation, not tailored individual results. This approach, meant to set realistic expectations, points to future research directions for creating personalized postoperative images, enhancing patient care and informed decision-making. Incorporating plastic surgeons’ feedback and comparing AI-generated images with actual surgical outcomes could significantly improve AI’s accuracy and utility in clinical settings. A balance in preference between AI and real postoperative images may indicate AI’s effectiveness in setting realistic patient expectations, highlighting the importance of aligning AI models with practical surgical results.

With an average processing time per image set of 56 ± 11.8 ms and total development costs of USD 321.60, this GAN model represents a cost-effective and rapid outcome simulator with potential clinical adoption. High-speed processing and prediction prevent time delays in pre-operative consultation while potentially increasing the clinic-to-operating-room conversion rates and reducing time to decision-making [13]. Moreover, the low-cost development process contrasts with the USD 12,264 that rhinoplasty patients are willing to pay per quality-adjusted life-year [14]. The fact that comparable outcome simulation models charge monthly fees of up to USD 556 further relatives our development costs. Finally, the minimal outlay required to program, train, and validate our GAN may help colleagues from low-income countries integrate our network into their pre-operative patient consultation process.

### Limitations

This study is not without limitations: Prolific users may not be assumed to make the best effort to actually determine the AI-generated versus real image since they are commonly paid per hour, meaning that they may have incentive to complete as many classification tasks as possible. Focusing on profile snapshots, the frontal view and the internal view, both essential for assessing airflow obstruction, were not included in the model development [15]. This approach relies on two-dimensional profile view images, although the frontal view is particularly important in rhinoplasty outcome simulations. This view has proven challenging in accurately representing nasal anatomy using existing technologies. Further studies should incorporate three-dimensional pre-operative simulation, as their utility for rhinoplasty is well documented [16]. While our algorithm represents a novel approach to AI-based outcome simulation in facial surgery (human evaluation panel, heterogeneous and large study population, cost-effectiveness, algorithm code publicly available), it should be noted that the concept of AI-based pre-operative simulations is not new to the field of facial surgery [17]. Future research may involve rhinoplasty experts to add more clinical expertise and experience to the evaluation panel. Additionally, the next research steps may present a second group of photographs to the participants, including standard morphing photographs generated by the surgeon and actual postoperative photographs. Future research may leverage commercial software to integrate the patient’s individual expectations into our GAN algorithm. Moreover, the additional use of electronic measurement software might have provided an additional perspective and should be used in upcoming studies. We included 1,015 female and 1,015 male rhinoplasty patients in this study. However, gender was determined based on online image database, Inc. patient information. To broaden the applicability, we aim to incorporate long-established rhinoplasty databases, such as Rhinobase, into future surgical outcome simulators [18]. However, it should be noted that the use of a large database with various outcome images of different rhinoplasty surgeons can also be regarded as a limitation: AI-generated outcomes from a varied rhinoplasty database may not reflect individual surgeon styles, limiting specificity. Tailored AI systems using a surgeon’s own images could improve accuracy. This distinction highlights the potential variability in AI training approaches. Incorporating plastic surgeons’ feedback and comparing AI-generated images with actual surgical outcomes could significantly improve AI’s accuracy and utility in clinical settings. A balance in preference between AI and real postoperative images may indicate AI’s effectiveness in setting realistic patient expectations, highlighting the importance of aligning AI models with practical surgical results.

Future trials are warranted to delve deeper into any gender differences and provide modifiable simulations. Such refinements may also help incorporate specific patient wishes as a pivotal step toward individualized outcome simulations. Lastly, non-matching pre-operative outcome simulations and postoperative results may cause litigation issues.

## Conclusion

We could show that GAN-based outcome simulators can generate images that resemble actual postoperative outcomes: The participants included in this study achieved an overall accuracy of 52.5% when identifying the AI-generated image. This method proved to be cost-efficient, utilizing minimal training data and rapid simulation capabilities.
